# Detection of Haplotypes Associated with Prenatal Death in Dairy Cattle and Identification of Deleterious Mutations in GART, SHBG and SLC37A2

**DOI:** 10.1371/journal.pone.0065550

**Published:** 2013-06-07

**Authors:** Sébastien Fritz, Aurelien Capitan, Anis Djari, Sabrina C. Rodriguez, Anne Barbat, Aurélia Baur, Cécile Grohs, Bernard Weiss, Mekki Boussaha, Diane Esquerré, Christophe Klopp, Dominique Rocha, Didier Boichard

**Affiliations:** 1 UNCEIA, Genetics Team, Paris, France; 2 INRA, UMR1313 Animal Genetics and Integrative Biology, Jouy-en-Josas, France; 3 INRA, Sigenae, UR875 Biométrie et Intelligence Artificielle, Castanet-Tolosan, France; 4 INRA, GeT Genomics Facility, UMR444 Laboratoire de Génétique Cellulaire, Castanet-Tolosan, France; Institut Jacques Monod, France

## Abstract

The regular decrease of female fertility over time is a major concern in modern dairy cattle industry. Only half of this decrease is explained by indirect response to selection on milk production, suggesting the existence of other factors such as embryonic lethal genetic defects. Genomic regions harboring recessive deleterious mutations were detected in three dairy cattle breeds by identifying frequent haplotypes (>1%) showing a deficit in homozygotes among Illumina Bovine 50k Beadchip haplotyping data from the French genomic selection database (47,878 Holstein, 16,833 Montbéliarde, and 11,466 Normande animals). Thirty-four candidate haplotypes (p<10^−4^) including previously reported regions associated with Brachyspina, CVM, HH1, and HH3 in Holstein breed were identified. Haplotype length varied from 1 to 4.8 Mb and frequencies from 1.7 up to 9%. A significant negative effect on calving rate, consistent in heifers and in lactating cows, was observed for 9 of these haplotypes in matings between carrier bulls and daughters of carrier sires, confirming their association with embryonic lethal mutations. Eight regions were further investigated using whole genome sequencing data from heterozygous bull carriers and control animals (45 animals in total). Six strong candidate causative mutations including polymorphisms previously reported in FANCI (Brachyspina), SLC35A3 (CVM), APAF1 (HH1) and three novel mutations with very damaging effect on the protein structure, according to SIFT and Polyphen-2, were detected in GART, SHBG and SLC37A2 genes. In conclusion, this study reveals a yet hidden consequence of the important inbreeding rate observed in intensively selected and specialized cattle breeds. Counter-selection of these mutations and management of matings will have positive consequences on female fertility in dairy cattle.

## Introduction

Most dairy cattle breeds are genetically small populations formed few centuries ago from a limited number of founders. In the last 60 years, their genetic pool was further reduced by the wide use of a limited number of elite sires via artificial insemination and strong selection on small number of traits. As a consequence, typical dairy cattle breeds display an average inbreeding rate around 1% per generation corresponding to an effective size of 50 [1], [2] and fifty percent of their gene pool are explained by only 10 to 20 ancestors. Such an inbreeding trend, associated to a corresponding increase in homozygosity, is favorable to the expression of recessive defects. Consequently nearly all dairy cattle breeds are characterized by segregation of genetic abnormalities and new emergences are regularly observed such as Bovine Leukocyte Adhesion Deficiency (BLAD [3]), Complex Vertebral Malformation (CVM, [4]), Syndactyly [5] or Brachyspina [6], [7] in Holstein.

In recent years, implementation of dedicated observatories in different countries (such as in Denmark [8] and in France [9]), and use of homozygosity mapping based on high density SNP genotyping data [10], [11] have proven very efficient tools enabling the detection of novel genetic defects and identification of associated mutations in a very short period of time with a limited number of cases. However, this process rely on the observation of clinical cases (i.e. on animals still alive up to birth and showing clinical signs) and except rare cases of late abortion (as for CVM or Brachyspina), most of the defects responsible for embryonic or fetal death are missed. Female fertility is yet a major concern in modern dairy industry. Calving rate is known to regularly decrease over time [12] and only half of this decrease is explained by indirect response to selection on milk production, suggesting the existence of other factors including embryonic lethal genetic defects.

In 2011, VanRaden et al. [13] proposed to detect them by searching a deficit in homozygous animals among the tens of thousands of individuals genotyped for genomic selection with high density SNP beadchips. Using this approach they successfully identified five regions in the Holstein, Jersey and Brown Swiss breeds and subsequently identified a strong candidate causative mutation for two of them: two nonsense mutations in APAF1 [14] and in CWC15 [15].

The purpose of this study was to detect similar recessive lethal regions in the French Holstein, Normande, and Montbéliarde dairy breeds and characterize them up to the identification of strong candidate causative mutations.

## Results and Discussion

### Screening for Haplotypes Displaying a Deficit in Homozygotes

Seventeen, 11 and 6 haplotypes with significant deficit in homozygotes (p<10^−4^) were found in Holstein, Montbéliarde, and Normande breeds, respectively (Table 1). In consistence with the important founder effect observed in dairy cattle, none of these chromosomal regions were shared between the three breeds studied.

**Table 1:** List of regions displaying significant deficit of homozygotes.

Breed	Name	BTA	Interval (UMD3.1 Mb)	Expected nb of homoz.	Observed nbof homoz.	Haplotypefreq. (%)	Chi^2^ test
HOL	BY	21	20.2–22.3	49	0	3.6	2.6E-12
HOL	HH1	5	61.4–66.2	18	0	2.6	2.2E-05
*HOL*	*HH2*	*1*	*93.0–97.4*	*14*	*3*	*1.7*	*3.3E-03*
HOL	HH3	8	94.0–96.5	21	0	2.5	4.6E-06
HOL	HH4	1	1.9–3.3	49	0	3.6	2.6E-12
HOL	HH5	3	45.8–47.6	68	24	3.9	9.5E-08
HOL	HH6	3	49.4–52.6	91	38	4.6	2.8E-08
HOL	HH7	6	51.6–52.6	202	100	6.9	7.1E-13
HOL	HH8	7	78.8–80.1	15	0	2.1	1.1E-04
HOL	HH9	10	74.8–77.0	40	13	2.9	2.0E-05
HOL	HH10	11	31.5–33.2	24	1	2.2	2.7E-06
HOL	HH11	12	2.0–3.6	75	35	3.2	3.9E-06
HOL	HH12	15	77.3–79.4	127	54	5.5	9.3E-11
HOL	HH13	18	56.4–58.4	32	5	3.7	1.8E-06
HOL	HH14	19	42.7–43.9	40	8	2.4	4.2E-07
HOL	HH15	20	58.5–59.6	68	25	2.7	1.8E-07
HOL	HH16	26	10.4–12.8	26	2	2.0	2.5E-06
HOL	HH17	26	24.9–26.0	33	5	1.8	1.1E-06
MON	MH1	19	27.6–29.4	131	0	9.0	2.5E-30
MON	MH2	29	27.9–29.1	80	1	7.0	1.0E-18
MON	MH3	2	31.5–32.8	39	9	5.1	1.6E-06
MON	MH4	4	52.0–53.2	21	1	3.5	1.3E-05
MON	MH5	6	73.3–74.4	122	14	7.1	1.4E-22
MON	MH6	7	80.1–81.7	21	3	2.6	8.6E-05
MON	MH7	9	84.6–86.4	126	21	7.1	8.4E-21
MON	MH8	13	76.4–77.6	26	3	3.5	6.5E-06
MON	MH9	20	24.2–25.7	33	6	2.6	2.6E-06
MON	MH10	24	22.9–24.6	26	0	2.5	3.4E-07
MON	MH11	24	33.4–34.6	159	29	7.2	6.4E-25
NOR	NH1	24	38.1–39.2	12	0	1.8	5.3E-04
NOR	NH2	1	145.7–146.8	49	14	3.8	5.7E-07
NOR	NH3	4	92.3–93.8	41	10	5.9	1.3E-06
NOR	NH4	6	37.7–38.9	38	12	5.2	2.5E-05
NOR	NH5	7	3.6–4.6	58	20	1.9	6.0E-07
NOR	NH6	15	59.8–61.1	45	17	1.9	3.0E-05

Homoz: homozygote, freq: frequency. Only regions with Chi-square test p-values lower than 10^−4^ are shown except HH2 (italic), previously reported by [13].

Haplotype length varied from 1 to 4.8 Mb and frequencies from 1.7 to 9%. The five most frequent values (higher than 7%) were observed in Montbéliarde, and the most significant one was MH1 located on chromosome 19, with no observed homozygous vs 121 expected. In most situations (26/34), the number of observed homozygous progeny per haplotype was not null, suggesting either incomplete linkage disequilibrium between the haplotype and a deleterious mutation or incomplete penetrance of this unknown mutation on the homozygous state.

For instance, this is the case for haplotype HH5, with 24 observed homozygous against 66 expected. Interestingly, a strong linkage disequilibrium was observed between this haplotype and the causative mutation for CVM located 2 Mb upstream (around 43.4 Mb on UMD3.1 genome assembly [16]) on bovine chromosome 3 (BTA03). Indeed, among 8359 genotyped bulls in the French population, 244 were CVM carriers and 209 of them (86%) also carried HH5. However, 225 out of 8115 CVM-free bulls also carried HH5. A similar pattern held for HH6 located 6 Mb downstream of CVM. Furthermore, analysis of BTA03 haplotyping data confirmed that all of the CVM-HH5 carriers (except three, likely explained by one CVM and two SNP genotyping errors) carry a long single haplotype including the causative mutation and the detected haplotype. The main reason is likely that the original mutation has occurred in a frequent haplotype, not detected in this study, but in strong linkage disequilibrium with a rarer distant haplotype (HH5 and HH6). This result indicates that the causative variants may lay several Mb outside the detected intervals.

In addition to CVM, two other regions corresponding to known genetic defects were also detected in Holstein: Brachyspina and Syndactyly on chromosomes 21 and 15, respectively. Syndactyly is not a lethal defect but obviously no affected animal was genotyped as a candidate for genomic selection, explaining the deficit in homozygous. HH1 and HH3, two of the three regions reported in Holstein [13] were also detected. The third one, HH2, was hardly detected (p = 0.003) and was rarer in the French population than in the North American one.

### Effect of these Haplotypes on Fertility

Conception rate was then analyzed according to the predicted genotype of the sire and maternal grandsire in order to detect haplotypes associated with embryonic lethality among the a priori unknown phenotypes which prevents homozygous animals to be candidate for genomic selection (table 2). A significant loss of fertility both in heifers and adult cows in matings at risk (between carrier bulls and daughters of carrier sires) was observed for nine out of the 34 haplotypes, confirming the segregation of embryonic lethal mutations in the population studied.

**Table 2:** Loss in calving rate in matings between carrier sires and daughters of carrier sires.

Breed	Haplotype name	Heifer matings at risk (nb)	Loss in heifercalving rate (%)	Cow matings atrisk (nb)	Loss in cow calving rate (%)
**HOL**	**BY**	**21386**	*−* 6.67***	**70918**	−4.25***
**HOL**	**HH1**	**9388**	*−* 9.89***	**38072**	−4.90***
*HOL*	*HH2*	*1844*	*−0.83*	*882*	−*4.30* *
**HOL**	**HH3**	**5281**	*−* 5.40***	**7315**	−5.54***
**HOL**	**HH4**	**31663**	*−* 5.80***	**71788**	−1.74***
**HOL**	**HH5**	**155220**	*−* 1.45***	**380262**	−2.17***
**HOL**	**HH6**	**171443**	*−* 1.91***	**466195**	−0.35***
HOL	HH7	140795	1.01	368630	1.29
HOL	HH8	2081	0.26	884	0.99
HOL	HH9	3626	0.32	3828	−0.20
HOL	HH10^a^	0	0.00	0	0.00
HOL	HH11	15379	−0.21	28230	−0.61
HOL	HH12	49096	1.09	115176	0.95
HOL	HH13	17273	−0.87*	32323	1.83
HOL	HH14	10403	−0.44	4688	1.54
HOL	HH15	3035	0.64	7617	0.99
HOL	HH16	3402	1.27	1419	0.66
HOL	HH17	5536	0.29	9225	1.31
**MON**	**MH1**	**145978**	−5.96***	**403695**	−4.84***
**MON**	**MH2**	**112585**	−5.26***	**207697**	−4.85***
MON	MH3	36413	−0.90**	80974	−0.36
MON	MH4	2387	−0.60	4351	2.07
MON	MH5	60511	−0.42	141238	−0.59***
MON	MH6	16005	−1.04*	24559	−0.54
MON	MH7	64255	−0.27	189756	−0.03
MON	MH8	23414	−1.54***	72369	−0.34
MON	MH9	11134	0.42	28875	2.52
MON	MH10	23471	−0.16	30320	0.99
MON	MH11^a^	0	0.00	0	0.00
NOR	NH1	7055	−4.00***	5061	0.18
NOR	NH2	12871	−1.90***	22922	0.81
NOR	NH3	27674	1.20	52185	−0.03
NOR	NH4	21087	0.26	42583	0.37
**NOR**	**NH5**	**57333**	−1.39***	**183521**	−0.78***
NOR	NH6	84491	−1.42***	194422	0.36

* p<0.05, **p<0.01 and ***p<0.001 versus control group (t-test). Haplotypes with significant negative effects on conception rate in both heifers and adult cows in matings at risk are shown in bold type. Nb: number. a: no mating at risk was observed between carriers of these haplotypes and daughters of carrier bulls during the period of time studied (see methods).

Estimates of fertility loss were close to expectations for only six regions (MH1, MH2, BY, HH1, HH3, HH4) under the assumption of complete lethality (i.e. between −4.75 and −6.25% according to average conception rate) whereas the other ones showed results below the expectations.

For example, the loss of fertility associated with HH5 and HH6 “CVM” haplotypes was only around −1.5% as a result of the combined effects of incomplete linkage disequilibrium and of the birth of stillborn calves. The latter example shows that regions with lower than expected negative effects on fertility must also be taken into consideration.

### Identification of Candidate Causative Mutations

Whole genome sequencing (WGS) data from 25 Holstein, 11 Montbéliarde and nine Normande bulls with important individual contribution to the actual genetic pool of their breed (from 1.1 to 10.8%) were subsequently investigated to identify the deleterious mutations associated with eight of these nine haplotypes (see methods). HH3 was investigated in the framework of the “1,000 bulls genomes” project and the causative mutation will be presented in the forthcoming “1,000 bulls genomes” publication (Hayes et al., submitted).

Filtering for mutations that were (a) located at+or –6 Mb from the detected haplotype (b) carried in the heterozygous state by the carrier bulls and (c) absent from the non carrier bulls from the three breeds yielded a small number of polymorphisms predicted to modify the amino acid sequence of a protein (Table 3). Then, investigation of the consequence of these mutations on the protein structure and function (according to SIFT [18] and Polyphen-2 [19]) yielded strong candidate causative mutations for all haplotypes but NH5.

**Table 3:** List of candidate mutations for embryonic lethal defects.

Hap.	Nb of carriers and sequence coverage	Chromosome and interval investigated	Polymorphism	Gene	Consequence
BY	2	BTA21	g.20562277C>T	MYADM ortholog	p.V103I
	11.3 and 35.6 x	14.2–28.3 Mb	g.20679305C>T	MYADM	p.E144K
			g.20898337G>C	MFGE8	p.R177G
			**g.21184870_21188198del**	**FANCI**	**p.V877Lfs27X** *
			g.21364778C>T	RHCG	p.V313I
			g.22172338C>T	MAN2A2	p.R889H
			g.27527731G>C	IL16	p.L16F
HH1	1	BTA5	g.58618466C>A	OR6C2	p.A307S
	25.4 x	55.4–72.2Mb	g.60292438G>A	TESPA1	p.R392H
			g.60390877G>A	NTN4	p.S409L
			**g.63150400C>T**	**APAF1**	**p.Q579X** *
HH4	2	BTA1	**g.1277227A>C**	**GART**	**p.N290T** *
	10.2 and 17.1 x	0–9.3 Mb	g.2490314G>A	MIS18A	p.C13Y*
			g.2498533G>A	MIS18A	p.G173E
			g.6169223T>C	BACH1	p.S446G
			g.6630979T>C	N6AMT1	p.K213R
HH5–HH6	3	BTA3	**g.43412427C>A**	**SLC35A3**	**p.V180F** *
	10.7, 12.0, 35.6 x	39.8–53.6 and 43.4–58.6 Mb	g.49942403T>C	BCAR3	Loss of an essential splice site at the end of exon 2*
MH1	2	BTA19	**g.27956790C>T**	**SHBG**	**p.Q52X** *
	13.7 and 39.2 x	21.6–35.4 Mb	g.28979780C>T	PIK3R5	p.R363H
MH2	2	BTA29	g.28297566C>G	OR8A1	p.L2V
	10.3 and 17.4 x	21.9–35.1 Mb	**g.28879810C>T**	**SLC37A2**	**p.R12X** *
NH5	1	BTA7	g.2317534C>A	GRM6	p.H421Q
	8.9 x	0–10.6 Mb	g.2586974C>T	BT.9012 (OBSCN fragment)	p.R295K
			g.2602279C>G	OBSCN	p.L549P
			g.2602280A>G	OBSCN	p.L549P
			g.3620610G>A	GMIP	p.R799Q

* : mutations with damaging predicted effect on the structure and function of the protein. In bold are shown the strongest candidate mutations for each haplotype. Mutation g.49942403T>C, predicted to affect BCAR3 splicing, was not retained as a plausible causative mutation since deficiency in BCAR3 is not lethal in mouse [17]. MIS18A p.C13Y substitution was also not retained as a plausible causative mutation as described in the manuscript.

Two mutations associated with HH4 were predicted to be deleterious: a A-to-C transversion at position 1277227 (g.1277227A>C) and a G-to-A transition at position 2490314 (g.2490314G>A) on BTA1.The first one causes the substitution of an asparagine in a putative manganese binding site [20] by a threonine in the GlycinAmide Ribonucleotide Transformylase protein (GART p.N290T). Interestingly, asparagine-290 is entirely conserved among eukaryotes supporting a key role of this amino acid in GART function (Figure S1A). Moreover GART is a trifunctional polypeptide required for de novo biosynthesis of purines [21] that are key components of molecules as important as DNA, RNA, ATP, etc. Therefore the loss of GART function is predicted to cause the death of the conceptuses in the very first stages after fertilization. Taken together these arguments strongly support a possible role of GART p.N290T in the loss of fertility observed in mating between HH4 carriers and daughters of HH4 carriers. The second mutation, g.2490314G>A, results in a cysteine-to-tyrosine substitution at residue 13 of the MIS18 kinetochore protein homolog A (S. pombe) (MIS18A p.C13Y). While homozygous knock-out of MIS18A is embryonic lethal in mouse [22], and while this mutation was predicted to be damaging according to SIFT and Polyphen on the human protein function, this mutation is less likely causative since residue 13 and neighboring amino acid sequence show only weak conservation across placental Mammals (Figure S1B). Moreover tyrosine-13 is the wild type variant in rabbit MIS18A protein.

Since the best candidate mutation (g.1277227A>C) was located 0.7 Mb outside HH4, a similar analysis to that implemented for CVM was carried out. Haplotypes of markers located from the beginning of BTA1 to the end of HH4 haplotype were analysed. Genotyping of 72 bull sires representing all the haplotypes with a frequency above 1% (and 74.8% of the haplotype diversity) revealed that mutation g.1277227A>C was only associated with HH4 whereas mutation g.2490314G>A was also associated with two other haplotypes (Document S1). Subsequent genotyping of 4 animals (out of 32) that were compound heterozygotes for haplotypes associated with g.2490314G>A confirmed their homozygosity and definitely eliminated this candidate. Finally, haplotype comparison revealed that a large haplotype (ranging from position 516404 to 2291153 on BTA1) encompassing allele g.1277227C was also segregating in French Holstein in association with the wild type allele g.1277227A and with another haplotype than HH4. Like for CVM, the incomplete linkage disequilibrium with the surrounding marker haplotype most probably explains why the candidate mutation lay outside of the detected haplotype.

Only one strong candidate causative mutation was identified for MH1 (g.27956790C>T on BTA19) and MH2 (g.28879810C>T on BTA29). The candidate mutation for MH1 (g.27956790C>T) is predicted to introduce a premature stop codon in the sex steroid-binding globulin resulting in the loss of approximately 90% of this protein (SHBG p.Q52X). SHBG is an androgen transporter that regulates the plasma metabolic clearance rate of steroid hormones by controlling their plasma concentration [23]. While, no animal model deficient in SHBG has been described so far, several examples of embryonic lethality associated with deficiency in enzymes involved in the synthesis of sex steroids or in steroids receptors have been reported in mouse [24]–[26] suggesting that these hormones play a substantial role during development. In addition, lack of Protein S, one of the two known paralogs of SHBG, causes embryonic lethal coagulopathy and vascular dysgenesis in mouse [27]. Taken together these observations support a possible connection between SHBG deficiency and fertility loss associated with MH1 haplotype.

The candidate mutation for MH2 (g.28879810C>T on BTA29) is also predicted to introduce a stop codon at the very beginning of the solute carrier family 37 member 2 protein (SLC37A2 p.R12X). The solute carrier superfamily comprises more than 300 members organized into 51 families [28] involved in the transmembrane transport of a wide range of solutes. Numerous examples of genetic defects caused by deficiency in solute carrier protein have been reported in human, mouse and in farm animals (including CVM) [29]. Their common feature is the altered trafficking of a particular type of molecule, leading to a lack or an excess of this solute in a given cell compartment. In a recent study Pan et al. have identified SLC37A2 as a phosphate-linked, glucose-6-phosphate antiporter [30]. Glucose-6-phosphate (G6P) is a key molecule in cellular energy metabolism and deficiency in several enzymes involved in G6P metabolism (G6PD1, GPI1, GCK) [31]–[33] is embryonic lethal in mouse. These arguments suggest that the SLC37A2 null mutation herein reported is the causative mutation associated with harmful haplotype MH2.

Genotyping of 18 Montbéliarde bulls representing all the haplotypes with a frequency above 1% (and 81.52 and 82.10% of the haplotype diversity respectively) showed that these mutations were exclusively associated with MH1 and MH2 haplotypes. Interestingly genotyping of the unique animal that was homozygous for MH2 revealed that it was in fact heterozygous for mutation g.28879810C>T on BTA29 indicating that the ancestral haplotype without the mutation is still segregating with a low frequency in the breed.

Finally, using a blind approach we identified previously reported causative mutations for Brachyspina (FANCI p.V877Lfs27X), CVM (SLC35A3 p.V180F) and HH1 (APAF1 p.Q579X) confirming the reliability of the approach implemented to identify the candidate causative mutation for embryonic lethal defects.

### Conclusion

Here we report four novel loci associated to embryonic lethal defects and three strong candidate causative mutations. This present results show that complex traits such as fertility can be partly decomposed in elementary loci with Mendelian inheritance. Their discovery is not more difficult than for a classical Mendelian defect and will benefit from the large sequencing effort of many influential ancestors in each breed.

This study is a data mining study which provides a number of results and requires functional validation to confirm the predicted effect of the herein reported candidate mutations. However, a rigorous validation of embryonic lethal defects will be very difficult to implement in large farm animals, because it requires many embryos at different development stages.

Even if these polymorphisms are only good candidates to date, arguments are strong enough to justify their use in selection to avoid mating at risk and decrease their frequency in the populations. However, it should be kept in mind that most of these haplotypes are at low frequency and are responsible only for a small part of fertility variance, as already noticed by VanRaden et al. [13]. In this context, gradual selection against these deleterious haplotypes is more recommended than a drastic elimination of all carriers, which would be a costly policy for a limited gain.

In parallel of this study on embryonic lethal, we will focus also on the other regions to understand their effect and the reason of the deficit in homozygotes. Investigations on live calves will be easier to carry out and more similar to usual study of genetic defects.

In conclusion, with large scale genotyping for genomic selection, a domestic population becomes an experimental design by itself and makes it possible to detect many genes of interest accurately. As a consequence, domestic species may become of high interest as a complementary model to discover and annotate gene functions.

## Materials and Methods

### Ethics Statement

Experiments reported in this work comply with the French National Institute for Agricultural Research (INRA) ethical guidelines. The genotype database is the result of the Cartofine, Amasgen and Lactoscan projects funded by the French National Research Agency (ANR) and ApisGene, and from genomic selection activity generated by the French breeding companies. The whole genome sequence data originated from the CartoSeq project funded by ANR and ApisGene. All the samples and data analyzed in the present study were obtained with the permission of breeders, breeding companies and by funding organizations.

### Animals and Genotyping Data

Genotyping data were extracted from the French database for dairy genomic selection. All animals included in this study were genotyped with the Illumina Bovine 50k Beadchip® and had their sire and their maternal grandsire genotyped with the same chip. In most cases, their dams were not genotyped. In total, this study included 47,878, 16,833, and 11,466 animals in the Holstein, Montbéliarde, and Normande breeds, respectively. Pedigree information on a least 2 generations was available for all these animals.

### Quality Control and Phasing

Genotypes were first processed according to the French genomic selection procedures [34]. Markers with minor allele frequency lower than 3% over the three breeds or deviating from Hardy Weinberg equilibrium (p<10^−4^) were discarded. Only autosomal markers with a confirmed position on UMD3.1 genome assembly were kept [16]. Finally, after editing, 43,582 markers were kept for the subsequent analyses. Phasing was performed with DualPhase, part of the PhaseBook software [35]. This software combines family information and linkage disequilibrium at the population level. Because sires and grand-sires were genotyped and family size was rather large, these phases were highly reliable.

### Genetic Analysis

The analysis was performed by haplotypes of n markers. In this study, n was set to 20, defining a sliding window of one to 1.5 megabases. Because cows were rarely genotyped, we considered the information from their sire (referred to as the maternal grandsire of the embryo in this study), their mated bull (referred to as the sire in this study), as well as the haplotypic frequency in the population. For a given window, haplotypes were listed and their frequencies in the maternal chromosomes were estimated by counting. To avoid undesirable effects of frequency variation over generations on detection results, animals without progeny were not included in the frequency estimation. Only haplotypes with a frequency higher than 1% were considered thereafter. The number of observed homozygous progeny O(k) was counted for each haplotype k and compared to its expectation E(k) under the null hypothesis of neutrality. This expectation was estimated from the genotype of the sire, the genotype of the maternal grandsire, and the frequency in the population. In order to make the analysis more robust to the estimation of haplotype frequency, counting included only sires with at least one copy of the haplotype studied.
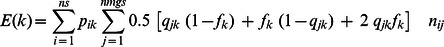
which simplifies to:



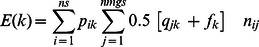
with ns being the number of sires of the conceptus (i.e. mating bulls of the cows), nmgs the number of maternal grandsires (cow’s sires), f_k_ the frequency of haplotype k, p_ik_ the transmission probability of haplotype k by sire i to his progeny (0.5 or 1 according to its genotype heterozygous carrier, or homozygous), and q_ik_ the transmission probability of haplotype k by grandsire j to his daughter dam of the progeny (0, 0.5 or 1 according to its genotype non carrier, heterozygous carrier, or homozygous), and n_ij_ the number of progeny with sire i and maternal grandsire j. Recombinations were omitted in this initial counting step. The final interval was defined by merging all windows with the same minimum number of homozygous products, from the first marker of the left window to the last marker of the right window.

In the few situations where the dams were also genotyped, the dam replaced the maternal grandsire in the formula as follows. In that situation, E(k) does not depend any more on haplotype frequency
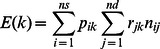
with nd being the number of dams and r_ik_ the transmission probability of haplotype k by the dam j, defined as q_ik_.

A recessive defect causing early mortality was suspected when O(k) was significantly lower than E(k). A chi square test was used to assess the significance of the difference between observed and expected results.

This analysis was conducted in the three breeds and over the 29 autosomal chromosomes with a window of 20 markers sliding by one marker at a time.

### Fertility Analysis

The effect of these regions was tested on fertility. Because these loci have recessive effect, the expected decrease in fertility of the daughters of a carrier bull reaches [0.5 (0.5+f_k_) f_k_ µ], with µ being the average conception rate, and is easily detectable only for frequent haplotypes. Therefore, a direct analysis of conception rate after individual artificial inseminations was carried out, with a model including the combination of the status of mated bulls with that of the sire of the cow. Conception rate was coded with a value of 1 when a calving was observed in an interval compatible with gestation length, 0 otherwise. The status (carrier or not carrier) was defined for 1,323 Montbéliarde bulls, 1,159 Normande bulls and 5,696 Holstein bulls born from 2000 and 2008. Only inseminations before 2010 with known status for both mating bull and sire of the cow were considered. A lethal defect during gestation is expected to lead to a decrease in conception rate close to −µ/8 in matings at risk. Mean conception rate varies from 40 to 55% according to breed and parity (virgin heifers vs lactating cows), leading to a loss in conception rate from 5 to 7% for the carrier x carrier combination. Tests were carried out separately for lactating cows and heifers, in order to get series of two independent results and also because the defects should have a stronger effect on heifers due to a higher average conception rate. Finally a t-test was used to determine any significant difference in conception rate between matings at risk and control groups.

### Whole Genome Sequencing, Read Mapping and Variant Calling

One to four paired-end libraries with a 250-bp insert size were generated by animals for a total of 25 Holstein, 11 Montbéliarde and nine Normande bulls using the Illumina TruSeq DNA Sample Prep Kit. Libraries were quantified with QPCRLibrary Quantification Kit (Agilent), controlled on a High Sensitivity DNA Chip (Agilent) and sequenced on one to four HiSeq 2000 lanes (Illumina) each with Illumina TruSeq V3 Kit (200 cycles). The 100-bp reads were mapped on the UMD3.1 bovine sequence assembly using the BWA tool [36]. Only reads with a unique mapping and a minimal quality of 30 were kept. PCR duplicates were filtered and a pileup of the mapped reads was created for each animal using SAMtools [37]. The average sequence depth of non-repetitive sequences ranged from 8.9 to 39.8 x. SNP and small indels (up to 10 bp) were detected with Genome Analysis Tool Kit 1.1–3 (GATK) [38] whereas discovery of larger indels was achieved with Pindel [39].

### Variant Filtering and Identification of Candidate Mutations

For each region, the carrier status of each sequenced founder bull was predicted based on its haplotypes. For HH3, no carrier was available in our pool of sequenced animals but one carrier was present in the “1,000 bull genomes” data set. The corresponding candidate mutation is presented in the forthcoming paper (Hayes et al, submitted). Therefore only 8 regions were presented here. Candidate mutations were searched in a large region including the whole haplotype and extended up to 6 Mb on each side. Only mutations found in the heterozygous state in carriers and absent in non carriers were considered. Then the consequences of the mutations on the protein structure were investigated (amino acid substitution, frame shift, gain or loss of stop codon, abnormal splicing). Missense mutations were retained when their predicted effect on the human ortholog was damaging according to both SIFT and Polyphen-2 softwares. When the wild type amino-acids were different between cattle and man, we firstly checked that the substitution of the human amino acid by the wild type cattle one was tolerated before testing the effect of the substitution of the human amino acid by the mutated cattle one. Across species conservation of the peptide sequence around the affected residue was also taken into account. Protein sequences were retrieved from Ensembl [40] and aligned using ClustalW version 2.0.1 software [41]. Finally, the potential effects of the selected mutations were assessed from the literature (animal models and protein known function).

### Validation of Candidate Mutations Identified in WGS Data from only One Carrier Bull

To confirm the existence of the novel candidate mutations identified in WGS data and to study their linkage with the surrounding haplotypes, 72 Holstein and 18 Montbéliarde key ancestors were subsequently genotyped by PCR-Sequencing for each mutation. These ancestors cover all haplotypes with a frequency above 1% and a large fraction of the total variability around the candidate mutations. These mutations included polymorphisms g.1277227A>C and g.2490314G>A on BTA01 (HH4), g.27956790C>T on BTA19 (MH1), and g.28879810C>T on BTA29 (MH2). PCR primers were designed from the UMD3.1 bovine genome assembly with Primer3 software [42]. The list of all the primers used in this study is available in Table S1. PCR reactions were performed using the Go-Taq Flexi (Promega) according to the manufacturer’s instructions on a Mastercycler pro thermocycler (Eppendorf). The resulting amplicons were purified and bidirectionally sequenced by Eurofins MWG (Germany) using conventional Sanger sequencing. Polymorphisms were detected with the NovoSNP software [43]. Sequencing electrophoregrams are presented in Figure S2.

## Supporting Information

Figure S1 Multispecies alignment of the GART (A) and MIS18A (B) protein sequences around the amino acid substitution p.N290T and p.C13Y, respectively. A) Cattle (Bta), chicken (Gga), anole lizard (Aca), coelacanth (Lch), xenopus (Xtr), Caenorhabditis elegans (Cel), Ciona intestinalis (Cin), fruitfly (Dme), black-legged tick (Isc), yeast (Sce), Sporisorium reilianum (Sre), Arabidopsis thaliana (Ath) and rice (Osa) protein sequences accession numbers in Ensembl are ENSBTAP00000012108, ENSGALP00000035973, ENSACAP00000004546, ENSLACP00000022410, ENSXETP00000030933, F38B6.4, ENSCINP00000015809, FBpp0079059, ISCW017017-PA, YGL234W, CBQ68240, AT1G09830.1 and LOC_Os12g09540.1, respectively. B) Cattle (Bta), Human (Hsa), rabbit (Ocu), panda (Ame), dog (Cfa), hyrax (Pca), pika (Opr), hedgehog (Eeu) and mouse (Mmu) protein sequences accession numbers in Ensembl are ENSBTAP00000022106, ENSP00000290130, ENSOCUP00000008087, ENSAMEP00000012611, ENSCAFP00000013042, ENSPCAP00000006502 ENSOPRP00000012255, ENSEEUP00000003845, ENSMUSP00000097150, respectively. Arrow indicates the wild type amino acid at the substitution site.
(TIF)

Figure S2 Sanger sequencing chromatograms confirming the existence of the candidate causative mutations identified in WGS sequence data.
(TIF)

Table S1 Details on the primers used in the present study.
(DOC)

Document S1 Details on the haplotypes in the HH4 region and their linkage with the candidate mutations. For each marker of the Illumina Bovine 50 k Beadchip, alleles A and B are denoted 1 and 2, respectively. Haplotypes HH4, MH1 and MH2 are underlined.
(XLS)
