# Supplementary figures and images for: Detection of Haplotypes Associated with Prenatal Death in Dairy Cattle and Identification of Deleterious Mutations in GART, SHBG and SLC37A2
Source: PLoS One. 2013 Jun 7;8(6):e65550. doi: 10.1371/journal.pone.0065550 (PMC3676330; doi:10.1371/journal.pone.0065550)

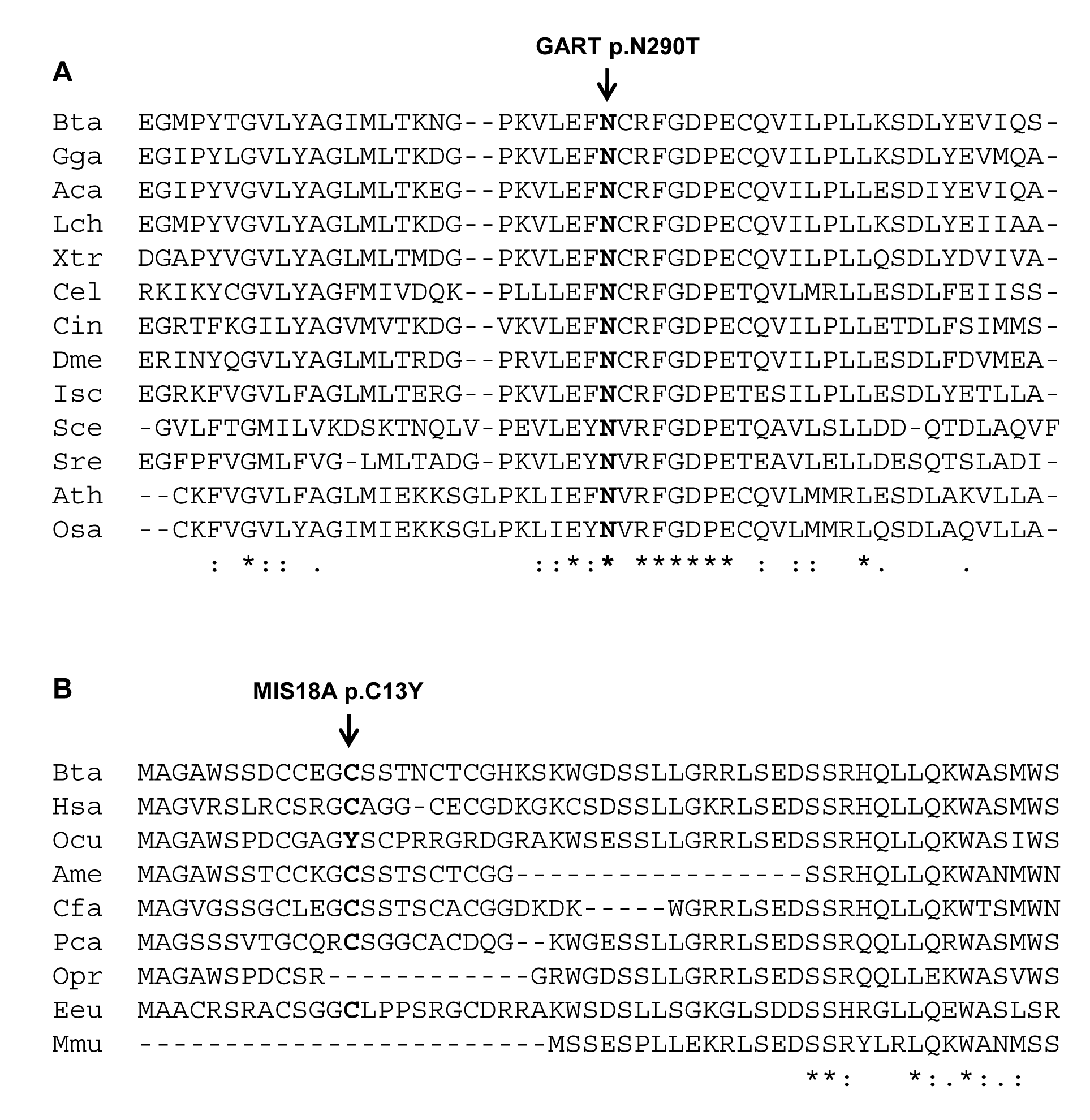

Supplement: Figure S1 — Multispecies alignment of the GART (A) and MIS18A (B) protein sequences around the amino acid substitution p.N290T and p.C13Y, respectively. A) Cattle (Bta), chicken (Gga), anole lizard (Aca), coelacanth (Lch), xenopus (Xtr), Caenorhabditis elegans (Cel), Ciona intestinalis (Cin), fruitfly (Dme), black-legged tick (Isc), yeast (Sce), Sporisorium reilianum (Sre), Arabidopsis thaliana (Ath) and rice (Osa) protein sequences accession numbers in Ensembl are ENSBTAP00000012108, ENSGALP00000035973, ENSACAP00000004546, ENSLACP00000022410, ENSXETP00000030933, F38B6.4, ENSCINP00000015809, FBpp0079059, ISCW017017-PA, YGL234W, CBQ68240, AT1G09830.1 and LOC_Os12g09540.1, respectively. B) Cattle (Bta), Human (Hsa), rabbit (Ocu), panda (Ame), dog (Cfa), hyrax (Pca), pika (Opr), hedgehog (Eeu) and mouse (Mmu) protein sequences accession numbers in Ensembl are ENSBTAP00000022106, ENSP00000290130, ENSOCUP00000008087, ENSAMEP00000012611, ENSCAFP00000013042, ENSPCAP00000006502 ENSOPRP00000012255, ENSEEUP00000003845, ENSMUSP00000097150, respectively. Arrow indicates the wild type amino acid at the substitution site. (TIF) [file pone.0065550.s001.tif]

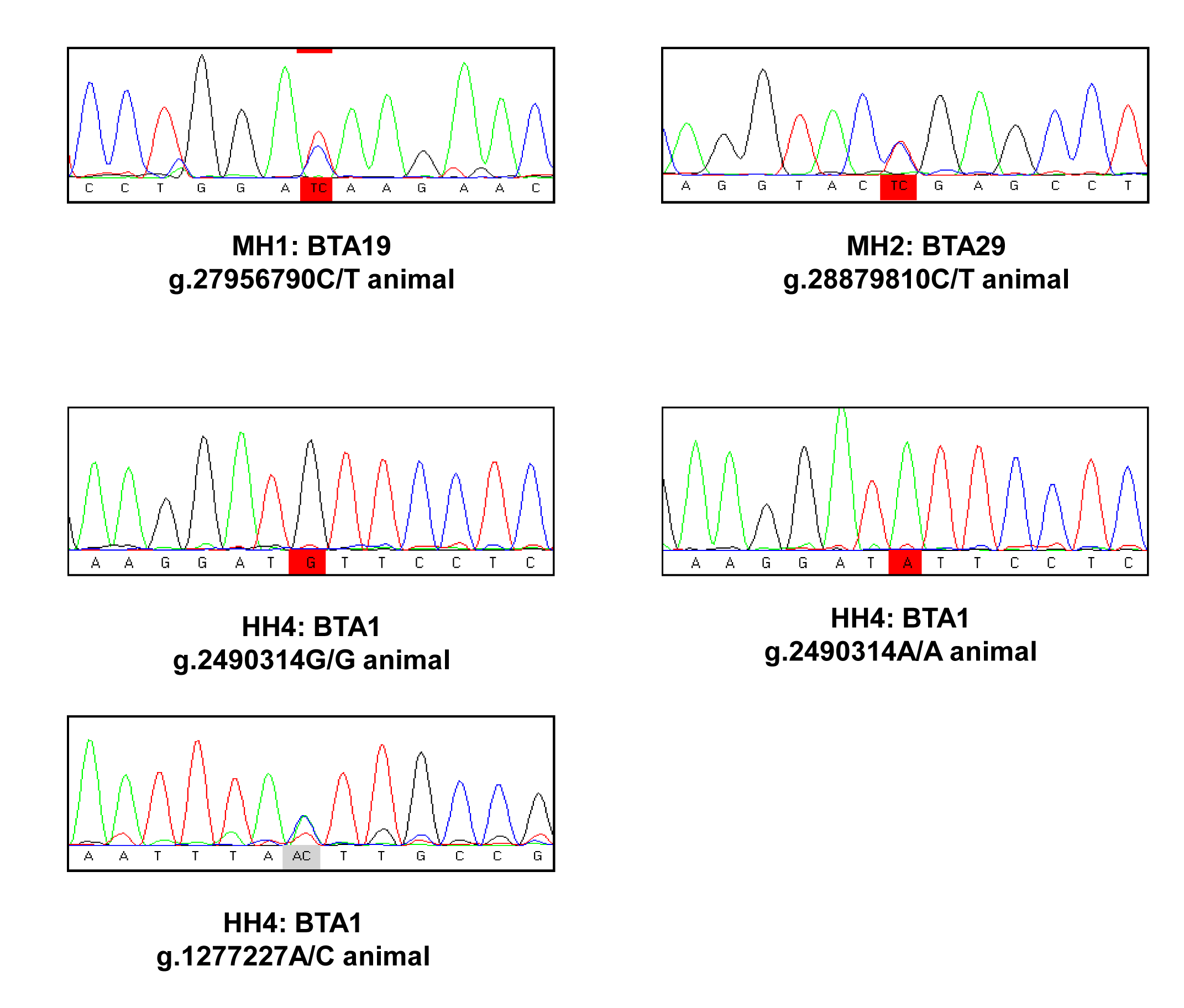

Supplement: Figure S2 — Sanger sequencing chromatograms confirming the existence of the candidate causative mutations identified in WGS sequence data. (TIF) [file pone.0065550.s002.tif]
