# Supplementary material for: Detection of Haplotypes Associated with Prenatal Death in Dairy Cattle and Identification of Deleterious Mutations in GART, SHBG and SLC37A2
Source: PLoS One. 2013 Jun 7;8(6):e65550. doi: 10.1371/journal.pone.0065550 (PMC3676330; doi:10.1371/journal.pone.0065550)
Supplement: Table S1 — Details on the primers used in the present study. (DOC) [file pone.0065550.s003.doc]

| **Haplotype** | **Polymorphism** | **Forward primer** | **Reverse primer** |
| --- | --- | --- | --- |
| MH1 | BTA19: g.27956790C>T | TGCTGCTGTTGCTACCACTT | AGCTGATGGAGAGAGGGTGA |
| MH2 | BTA29: g.28879810C>T | AGAACGAGCACAGGTGGACT | CCTGGGAAAAAGAACTGCTG |
| HH4_GART | BTA1: g.1277227A>C | AAGTGAAGTTGCCCAGTCGT | AAGTGCAGAGCAAGCCATCT |
| HH4_MIS18A | BTA1: g.2490314G>A | TGCGAGCAAATGTCCTACAG | AGACCCTGAGTTGCTCCAAA |
